# Prevalence of Ocular *Chlamydia trachomatis* Infection in Amhara Region, Ethiopia, after 8 Years of Trachoma Control Interventions

**DOI:** 10.4269/ajtmh.22-0535

**Published:** 2023-01-09

**Authors:** Scott D. Nash, Ambahun Chernet, Paul Weiss, Andrew W. Nute, Mulat Zerihun, Eshetu Sata, Demelash Gessese, Kimberly A. Jensen, Zebene Ayele, Berhanu Melak, Taye Zeru, Abdulkerim Mengistu, Adisu Abebe, Fikre Seife, Zerihun Tadesse, E. Kelly Callahan

**Affiliations:** ^1^ Trachoma Control Program, The Carter Center, Atlanta, Georgia;; ^2^ Trachoma Control Program, The Carter Center, Addis Ababa, Ethiopia;; ^3^ Department of Biostatistics and Bioinformatics, Emory University, Atlanta, Georgia;; ^4^ Amhara Public Health Institute, Bahir, Dar, Ethiopia;; ^5^ Amhara Regional Health Bureau, Bahir Dar, Ethiopia;; ^6^ Ministry of Health, Addis Ababa, Ethiopia

## Abstract

Although trachoma mass drug administration (MDA) programs target ocular *Chlamydia trachomatis*, the global trachoma control program does not monitor infection as a measure of impact but instead relies on monitoring clinical indicators. This study aimed to monitor the prevalence of ocular *C. trachomatis* among a population-based sample of children ages 1–5 years throughout Amhara, Ethiopia, a region that has received approximately 8 years of annual MDA as part of trachoma control. Between 2014 and 2021, trachoma impact surveys and surveillance surveys were conducted in all 156 districts of Amhara using a multistage cluster randomized methodology. Certified graders assessed individuals ages ≥ 1 year for trachomatous inflammation-follicular (TF), and a random subset of children ages 1–5 years also provided a conjunctival swab. Polymerase chain reaction was used to test for *C. trachomatis*. A total of 28,410 conjunctival swabs were collected from children ages 1–5 years across Amhara. The regional *C. trachomatis* infection prevalence was 4.7% (95% uncertainty interval: 4.3–5.1%). Infection was detected in all 10 zones of the region and ranged from 0.2% in Awi Zone to 11.9% in Waghemra Zone. Infection was detected in 17 (26%) districts with a TF prevalence < 10% and in 7 (21%) districts with a TF prevalence < 5%. Through programmatic monitoring of *C. trachomatis* infection, this study demonstrated that considerable infection remained throughout Amhara despite approximately 8 years of trachoma interventions and that enhanced interventions such as more frequent than annual MDA will be needed if elimination thresholds are to be reached.

## INTRODUCTION

Azithromycin, an antibiotic shown to be effective against ocular *Chlamydia trachomatis* infection, is the primary medication used in mass drug administration (MDA) for trachoma.^1^ Although MDA programs target *C. trachomatis*, the global trachoma program does not monitor infection as a measure of impact but instead relies on monitoring clinical indicators of trachoma. Clinical indicators have been shown to overestimate infection once MDA programs have begun and, as trachoma prevalence decreases, it is difficult to find clinical cases to reliably train survey graders.^2^^,^^3^ In-country, high-throughput testing on commercial platforms may be one solution for programs to monitor programmatic impact on *C. trachomatis* prevalence directly.

Starting in 2007, the Trachoma Control Program in Amhara Region, Ethiopia, has been at scale with the WHO-endorsed surgery, antibiotics, facial cleanliness, and environmental improvement (SAFE) strategy. This includes annual MDA with azithromycin, school- and community-based health education, and advocacy for latrine building, among other interventions.^4^ However, region-wide surveys conducted between 2011 and 2015, after 5 years of interventions, demonstrated that trachoma remained highly endemic in most districts, as measured by the clinical indicator trachomatous inflammation-follicular (TF).^5^ Furthermore, it was determined that the regional prevalence of *C. trachomatis* was 6% and that infection remained throughout all 10 administrative zones of Amhara, with one zone reaching a prevalence of nearly 20%.^2^

More recently, a second round of surveys was conducted to measure the impact of approximately 8 years of interventions across the region. Those surveys demonstrated that 72% of districts were still trachoma endemic.^4^ During this second round of surveys (2014–2021), the program continued to collect conjunctival swabs from a population-based sample of children ages 1–5 years throughout the entire Amhara region.

The aim of this study was to detail the ocular *C. trachomatis* infection prevalence in Amhara after approximately 8 years of SAFE interventions. Further, infection prevalence was compared cross-sectionally to the clinical indicators TF and trachomatous inflammation-intense (TI), and to *C. trachomatis* prevalence from the first round of surveys.

## MATERIALS AND METHODS

### Ethical considerations.

Survey protocols were reviewed and approved by institutional review boards at Emory University (Protocol 079-2006) and the Amhara Regional Health Bureau. For surveys conducted between 2017 and 2020, protocols were also reviewed by Tropical Data (https://www.tropicaldata.org/). Verbal informed consent and assent were obtained and recorded from all individuals examined for trachoma.

### Setting and timeline.

Amhara is administratively divided into 156 districts (locally known as “woredas”), which themselves make up 10 administrative zones. Because the SAFE strategy was scaled up over a period of 5 years, it took approximately 5 years to survey all districts for impact. The first round of impact surveys was conducted between 2011 and 2015 and included conjunctival swabbing for *C. trachomatis* infection.^2^^,^^5^ Based on the results of these surveys, SAFE interventions continued throughout the region. Between 2014 and 2021, all districts in Amhara were surveyed again. The median number of years of A, F, and E interventions, including annual MDA received prior to these surveys, was 8 years (interquartile range [IQR]: 8–10 years). In each year prior to these surveys, the program delivered on average 14 million doses of antibiotic with administrative coverage around 90%, had enrolled an average of 8,000 schools in a school trachoma hygiene program, and advocated for the building of approximately 500,000 new household latrines.^4^ This report details the results of surveys conducted within this 8-year time frame in those 156 districts. Each district is represented just once in this report.

### Survey methodology.

Between 2014 and 2021, population-based trachoma impact surveys (TISs) and trachoma surveillance surveys (TSSs) were conducted in all districts of Amhara designed to assess the prevalence of TF among children ages 1–9 years. As described in previous reports, a multistage cluster randomized methodology was used to estimate the prevalence in each district.^4^^,^^5^ In the first stage of selection, clusters (communities) were selected from a total list of communities using a population proportional to estimated size method. In the second stage, a modified segmentation approach was used to select a random segment within each cluster. All households within each segment were then surveyed. All individuals ages ≥ 1 year within selected households were enumerated, and all consenting individuals were examined for trachoma.

During the cluster selection process, approximately half of the selected clusters from each district were chosen randomly for the additional swab collection and then, within each cluster, the first 25 children ages 1–5 years encountered within selected households by survey teams were selected for swabbing.^2^ To estimate the *C. trachomatis* prevalence at the zonal level among children in this age range, a prevalence level of 4 ± 2%, an α error of 0.5, and a design effect of 3 were assumed.^2^ After further assuming a 10% nonresponse rate, 1,217 children per zone were targeted for swab collection.

### Data collection.

Prior to each survey round, trachoma graders participated in a well-characterized training program.^4^^,^^5^ Graders with an agreement of κ ≥ 0.7 compared with grader trainers on a field reliability examination of 50 conjunctivae were certified to participate in trachoma surveys. Consenting individuals had both their conjunctivae examined for signs of TF and TI as defined by the WHO simplified grading scheme.^6^ Examination was conducted using a 2.5× ocular loupe and a flashlight if needed. Individuals identified with TF and/or TI were offered treatment with tetracycline eye ointment per WHO recommendations.

### Conjunctival swabbing.

After grading clinical signs, the grader donned powder-free latex gloves and swabbed the conjunctivae three times with a polyester-tipped swab (Fisher Scientific, Waltham, MA), rotating 120° between each pass.^2^ With the help of a gloved “tuber,” swabs were placed dry into a 2.0-mL tube, labeled, and stored on ice while in the field. Swabs were transported to the laboratory on ice and then stored at −20°C until testing. To monitor contamination, graders also performed an “air swab” by passing a swab 1 inch in front of the conjunctivae of a randomly selected 5% of swabbed children. These air swabs were stored and transported in an identical manner as conjunctival swabs.

### Laboratory methodology.

Laboratory testing was performed at the Amhara Trachoma Molecular Laboratory at the Amhara Public Health Research Institute in Bahir Dar, Ethiopia. Conjunctival swabs from each district were randomized and pooled, five samples per pool.^2^ Laboratory technicians were masked to the district of origin and trachoma status of sample providers. Pools were tested using Abbott’s RealTime polymerase chain reaction (PCR) assay (Abbott Molecular, Des Plaines, IL) on the Abbott m2000 system to detect *C. trachomatis* DNA as described previously.^2^ Each plate on the m2000 can hold 96 pools, and thus up to 480 samples. Air swabs were pooled and handled in a similar manner to conjunctival swabs throughout the testing process. Pools with equivocal results were retested and, if equivocal on the second run, the individual samples in the pool were tested. If a district’s pooled prevalence was ≥ 80%, all samples from that district were repooled randomly into pools of three.^7^ Two (1.3%) of the 156 districts had a pooled prevalence high enough to warrant repooling.

As described in previous reports, strict laboratory quality control (QC) procedures were maintained throughout the project.^2^^,^^8^ The laboratory conducted biannual masked testing of standard QC panels (20 negative and 20 positive samples) prepared externally. The laboratory also performed regular testing for amplicon contamination. Annual preventative maintenance was performed by a local Abbott technician. Each year approximately 10% of tested pools, both positive and negative, were sent for repeat testing on an m2000 system at a laboratory in the United States.

### Statistical analysis.

*Chlamydia trachomatis* was defined as present if *C. trachomatis* DNA was detected in a pool. District infection prevalence was estimated from the pooled prevalence as the number of positive individual samples most likely to have resulted in observed pooled results.^7^ Regional-level and zonal-level estimates (10 zones total) were weighted by the inverse of the district-specific selection probability. Uncertainty intervals (UIs) around the weighted estimates were built using 1,000 random draws from a population where the zone prevalence estimates were centered around the observed values and then were allowed to fluctuate based on external measurements of prevalence rate variability in the districts. Within-zone variability was derived by an ANOVA-based estimate of prevalence variability to control for unequally sized zones. In this report, we present bootstrap UIs using the within-cluster variability estimates produced by the ANOVA approach.^9^

Between 2014 and 2017, district prevalences of TF and TI were determined using weights calculated as the inverse of the sampling probability of both stages of sampling.^4^ Starting in 2017, TF and TI were estimated by first age-adjusting cluster-level data into 1-year age bands based on the Ethiopian 2007 national census population structure and then taking the mean of the cluster prevalence estimates as the district prevalence.^4^ Due to a lower level of statistical precision at the district level, estimates at that level were presented either in aggregate or categorized. Pearson correlation coefficients were used to elucidate the relationships between district-level *C. trachomatis* prevalence among children ages 1–5 years and TF and TI prevalence among children ages 1–9 years.

## RESULTS

Between 2014 and 2021, 28,410 conjunctival swabs were collected from a population-based sample of children ages 1–5 years across 156 districts in Amhara. Among the 156 district surveys, 149 were TIS and 7 were TSS. The median number of swabs per district was 153 swabs, IQR: 111–193 swabs. These swabs were pooled in the laboratory to form 5,842 pools. The median number of pools per district was 31 pools (IQR: 23–39 pools).

Results of routine QC showed 100% (20/20) concordance for testing QC control panels each round and 0–5% contamination from routine amplicon monitoring over this study period. For pools tested externally in the United States, the following agreement was obtained by year: 2017: 97% (184/189); 2018 spring: 98% (355/363); 2018 fall: 100% (178/178); 2020: 99% (273/275); and 2021: 99% (307/309). Pools with discordant results were nearly all low-level positives (delta cycle range: 0.08–3.76). For field control air swabs, there were two positive pools and one “indeterminant” pool over this 8-year period. A retest of the individual samples in each pool found two positive samples and one sample that remained indeterminate. Thus, 0.08% (2/2,461) of air swabs could be considered contaminated.

**Figure 1.**
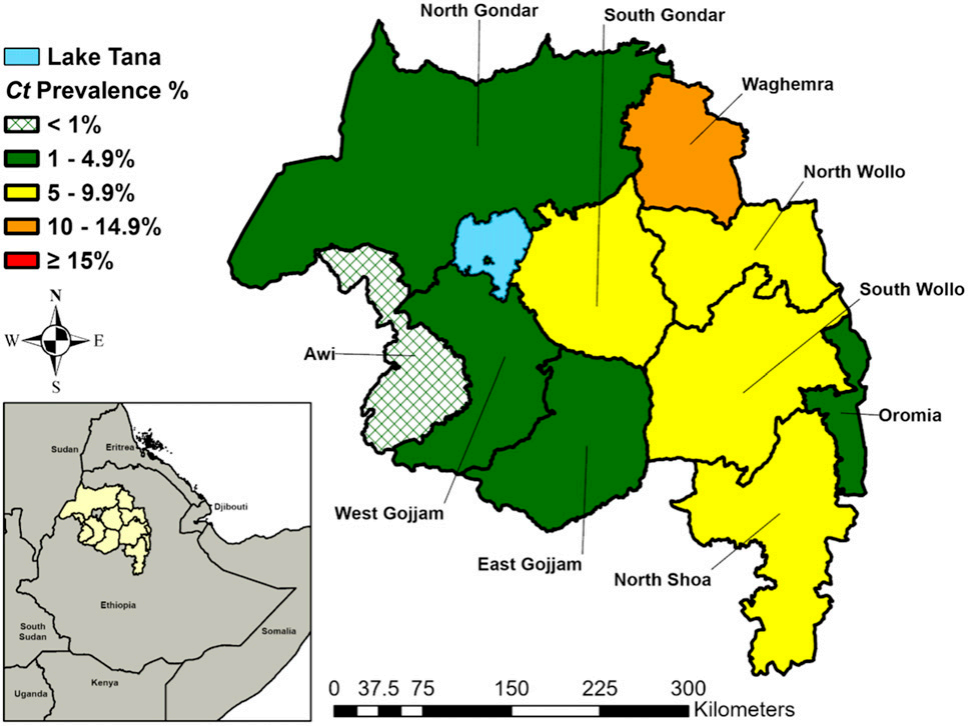
Zonal-level prevalence of ocular *Chlamydia trachomatis* infection among children ages 1–5 years, Amhara, Ethiopia, 2014–2021. *Ct* = *C. trachomatis*.

**Figure 2.**
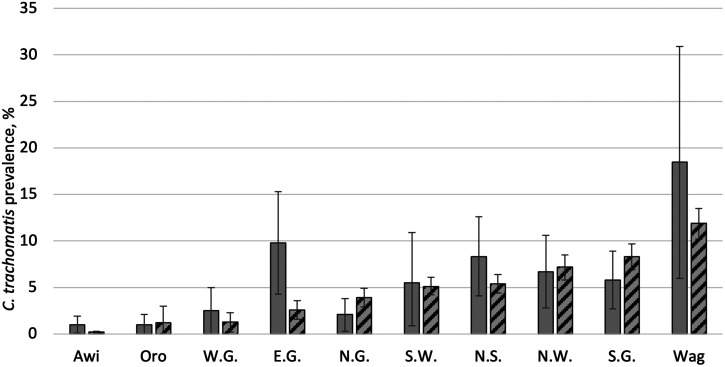
Prevalence of ocular *Chlamydia trachomatis* infection by zone in Amhara, Ethiopia, 2011–2015 after approximately 5 years of interventions and 2014–2021 after approximately 8 years of interventions. Solid bars represent the first round of surveys, 2011–2015. Striped bars represent the second round of surveys, 2014–2021. E.G. = East Gojjam; N.G. = North Gondar; N.S. = North Shoa; N.W. = North Wollo; Oro = Oromia; S.G. = South Gondar; S.W. = South Wollo; W.G. = West Gojjam; Wag = Waghemra. Bars represent 95% uncertainty intervals.

The regional *C. trachomatis* infection prevalence among children ages 1–5 years was 4.7% (95% UI: 4.3–5.1%) (Table 1). *C. trachomatis* infection was detected in all 10 zones (Figure 1) and ranged from 0.2% (95% UI: 0.1–0.3%) in Awi Zone to 11.9% (95% UI: 10.2–13.5%) in Waghemra Zone. Although 5 of 10 zones had a lower *C. trachomatis* infection prevalence at the second survey round (2014–2021) compared with the first survey round (2011–2015), UIs on each survey’s estimate overlapped for all zones except East Gojjam (Figure 2). The largest prevalence decreases were observed in East Gojjam Zone (prevalence difference: −7.2%) and Waghemra Zone (prevalence difference: −6.6%).

**Table 1:** Sample size of ocular *Chlamydia trachomatis* surveys, Amhara, Ethiopia, 2014–2021

Zone	Number of districts	Number of clusters	Number of pools	Number of swabs	*C. trachomatis* infection prevalence (%) (95% UI)	District prevalence range % (min–max)
Awi	11	90	400	1,991	0.2 (0.1–0.3)	0–0.5
East Gojjam	19	296	844	4,169	2.6 (1.6–3.6)	0–11.6
North Gondar	23	238	876	4,324	3.9 (2.9–4.9)	0–12.1
North Shoa	24	302	1,012	4,683	5.4 (4.4–6.4)	0–34.4
North Wollo	14	112	360	1,693	7.2 (5.8–8.5)	0–27.5
Oromia	7	58	205	1,016	1.2 (0.0–3.0)	0–3.0
South Gondar	12	95	420	2,058	8.3 (6.9–9.7)	0–26.1
South Wollo	22	290	946	4,633	5.1 (4.2–6.1)	0–22.1
West Gojjam	16	112	524	2,576	1.3 (0.2–2.3)	0–12.3
Waghemra	8	72	255	1,267	11.9 (10.2–13.5)	0–23.6
Amhara Region	156	1,665	5,842	28,410	4.7 (4.3–5.1)	0–34.4

UI = uncertainty interval.

**Figure 3.**
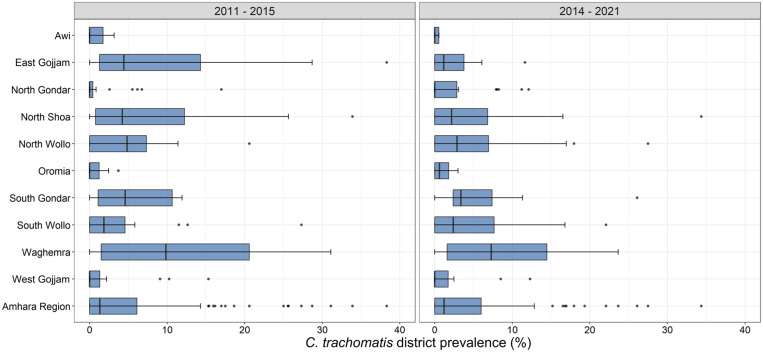
Prevalence of ocular *Chlamydia trachomatis* infection by district in Amhara, Ethiopia, 2011–2015 and 2014–2021. Box plots represent the median, 25% and 75% quartiles, and range. Points represent outliers.

The number of districts with at least one sample positive for *C. trachomatis* was 94/156 (60.3%). Among districts with infection detected, the infection prevalence ranged from 0.2% to 34.4% (Figure 3). In the first round of surveys, 8/150 (5.3%) had a *C. trachomatis* prevalence > 20%, compared with 5/156 (3.2%) in the second survey round.

**Figure 4.**
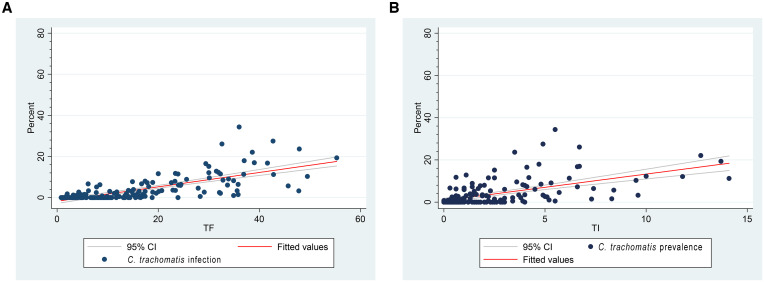
Correlation between district-level *Chlamydia trachomatis* infection (ages 1–5 years) and (**A**) TF (ages 1–9 years) and (**B**) TI (ages 1–9 years), Amhara, Ethiopia, 2014–2021.

As part of these surveys, 105,402 children ages 1–9 years were examined for TF and TI. The median district TF prevalence was 13.4% (IQR: 5.6–23.9%) and the median TI prevalence was 1.5% (IQR: 0.6–3.6%). The correlation between district-level *C. trachomatis* infection prevalence and the prevalence of clinical signs of trachoma among children ages 1–9 years was high; TF: *r* = 0.72, *P* < 0.0001; TI: *r* = 0.57, *P* < 0.0001 (Figure 4). Correlations between *C. trachomatis* infection and TF were most strongly observed in districts with ≥ 10% TF (*r* = 0.66, *P* < 0.0001) than when compared with districts with < 10% TF (*r* = 0.19, *P* = 0.14).

**Figure 5.**
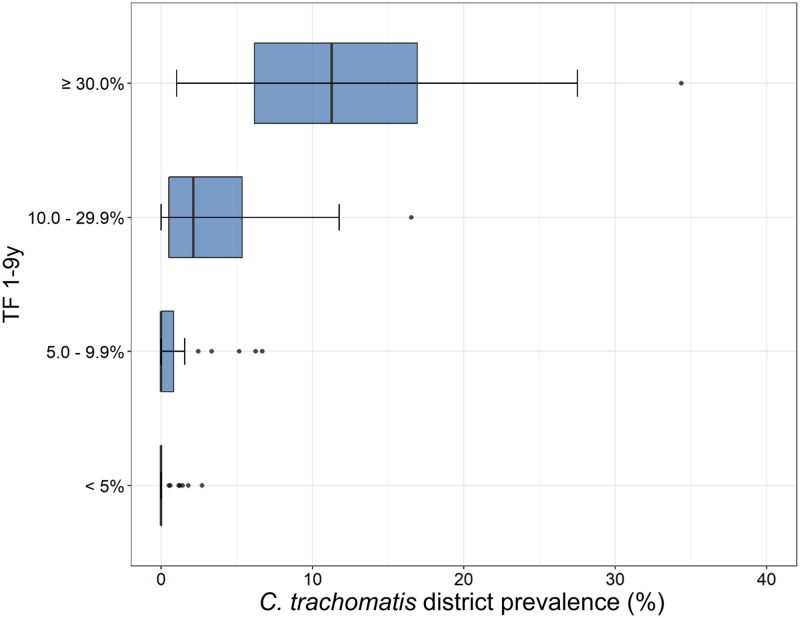
District-level *Chlamydia trachomatis* infection prevalence by WHO TF categories, Amhara, Ethiopia, 2014–2021. Box plots represent the median, 25% and 75% quartiles, and range. Points represent outliers.

**Figure 6.**
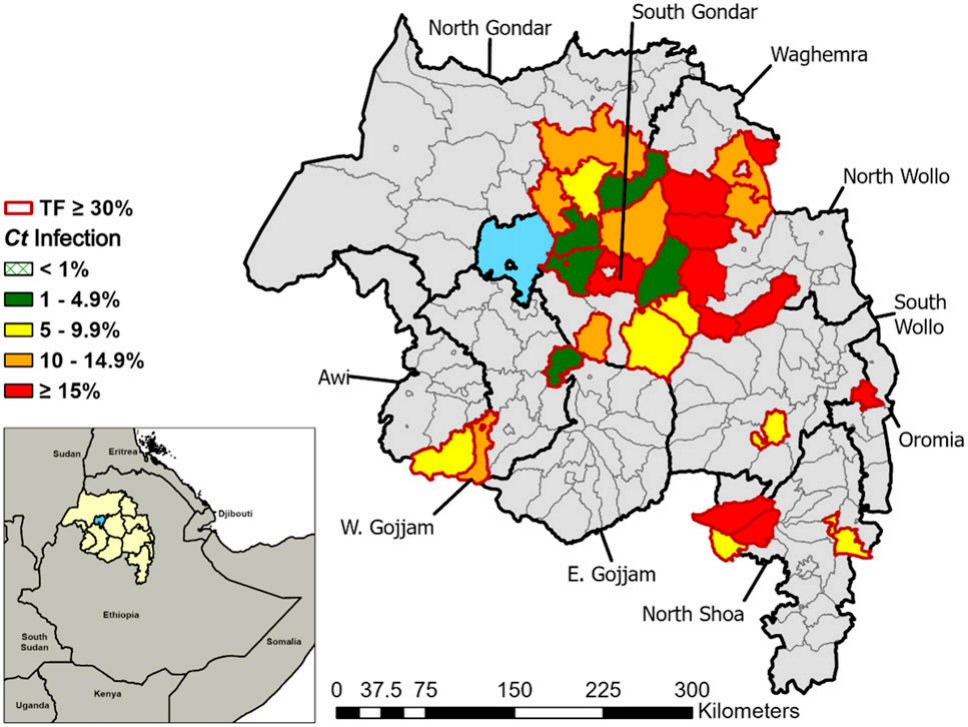
Ocular *Chlamydia trachomatis* infection within districts with TF > 30%, Amhara, Ethiopia, 2014–2021. *Ct* = *C. trachomatis*.

District-level *C. trachomatis* prevalence increased with each WHO category of TF prevalence (Figure 5). Every district with a TF prevalence ≥ 30% (*N* = 30) had infection detected, with a median prevalence of 11.3% (IQR: 6.1–17.0%). These districts with persistently high TF and *C. trachomatis* were generally found in the northeast and central parts of the region (Figure 6). Among the districts with a TF prevalence of < 10%, 17/65 (26%) had infection detected, with a median prevalence of 1.5% (IQR: 0.8–2.7%). Among districts with a TF prevalence of < 5%, 7/34 (21%) had infection detected, with a median of 1.2% (IQR: 0.6–1.8%). Lastly, among the seven TSSs conducted during this period, 2/7 (29%) had detectable infection, range 1.2–1.4%. All seven of the TSSs had a TF prevalence of < 5%.

## DISCUSSION

After approximately 8 years of SAFE interventions including MDA, a considerable *C. trachomatis* infection burden remains in Amhara. These data support the clinical trachoma results presented by Sata et al.^4^ that trachoma remains endemic throughout the region. The addition of infection monitoring at the programmatic scale further helped to refine which districts were experiencing persistent *C. trachomatis* transmission despite comprehensive interventions. With this much infection still present throughout the region, enhanced interventions will be needed to speed up progress toward trachoma elimination as a public health problem in Amhara.^4^

Since early in the program, it was observed that the trachoma prevalence in some districts in Amhara was not responding as quickly as expected to SAFE interventions. Reports of impact surveys conducted in the region demonstrated that some districts continued to have persistently hyperendemic levels of TF.^4^^,^^5^^,^^10^ These results were further supported by studies employing more objective methods such as PCR and serological testing of infection.^11^^,^^12^ Operational research conducted within these districts has examined MDA coverage in Amhara, as well as the potential contributions of antimicrobial resistance, water, sanitation, and hygiene characteristics, and untreated infants toward persistently high trachoma.^13^^–^^16^ In this report, districts considered hyperendemic (TF > 30%) for trachoma after 8 years of interventions had an average *C. trachomatis* prevalence of > 10%. The continued presence of *C. trachomatis* infection in the face of MDA pressure has also been observed in randomized trials conducted in Amhara. In a trial conducted in Waghemra Zone, where *C. trachomatis* prevalence was the highest in this study, 8 years of annual MDA resulted in a remaining community-level infection prevalence of approximately 15%.^17^ In East Gojjam Zone, although 7 years of high-coverage (> 90%) annual MDA resulted in reductions in community-level infection prevalence, a *C. trachomatis* infection prevalence of 9.9% remained.^18^ Recent modeling has further suggested that continued use of an annual MDA treatment program is unlikely to push these persistently endemic districts to the elimination threshold by the year 2030.^19^ In response, the global trachoma program has begun to consider increased programmatic flexibility in dealing with districts slow to reach elimination thresholds. An enhanced antibiotic treatment regimen characterized by more frequent rounds and/or targeted rounds to children along with a continued focus on F and E interventions is clearly warranted for persistent trachoma as observed in Amhara.

Infection prevalence was much lower in districts closer to the elimination threshold. Although there was a strong district-level correlation between TF and *C. trachomatis* infection overall in Amhara (0.72), this correlation was much lower (0.19) once the TF prevalence was < 10%. This is likely driven by the fact that only a quarter of districts with TF < 10% had detectable infection and, among those, the prevalence on average was < 1%. These findings suggest that among districts with a TF prevalence between 5% and 10%, ongoing *C. trachomatis* transmission may not be enough of a threat to warrant MDA. Studies are ongoing to determine whether halting MDA in districts such as these may lead to similar results as continuing MDA.^20^ The development of useful district-level *C. trachomatis* thresholds, likely through longitudinal studies, serial surveys, or forecasting models, would be a key step for wider use of infection monitoring within the global program. Two of the districts that had a TSS had detectable infection, even though they had remained below threshold for more than 2 years since MDA. A district obtaining a favorable TSS result (< 5% TF) with concurrent *C. trachomatis* infection has previously been reported within the region.^8^ Although it is difficult to know the importance of low levels of infection in this setting, these districts should be considered for longer-term postelimination monitoring to ensure sustainability of elimination.

Estimating the prevalence of *C. trachomatis* infection across the entire region of Amhara did require extra investment and logistical considerations compared with standard trachoma surveys. To collect swabs, an extra person serving as a tuber was added to each survey team. Although this helped ensure the quality of the sample collection, as demonstrated by the low contamination rate, it did add to the cost of training and field collection. The program also incurred additional supply costs from the swabs, tubes, and boxes, as well as the costs associated with keeping the samples cold throughout the process. Cost estimates have been generated for swab collection in the clinical trial setting; however, more work is needed to understand these costs in the programmatic setting.^21^ This study only assessed infection among children ages 1–5 years, and therefore infection among older children and adults was missed. This age group was chosen, however, as the youngest children tend to serve as a reservoir of infection for communities.^22^ This study was designed with adequate sample size for precise zonal-level estimates to aid in comparison with previous work in the region and because swabbing in all clusters in all 156 districts was cost prohibitive.^2^ Although district-level sampling was done in an unbiased way, there was likely a lack of precision in district estimates due to the smaller sample size. Future work in Amhara should target districts of programmatic relevance with surveys designed to provide more robust *C. trachomatis* prevalence estimates.

The use of high-throughput methods at regional laboratories could make infection prevalence a feasible, objective programmatic indicator for trachoma. The Trachoma Control Program in Amhara has monitored *C. trachomatis* infection prevalence regionwide alongside the clinical signs of trachoma for a period of 11 years. Over this time period, all samples were tested within a regional laboratory in Amhara with an excellent QC profile.^2^ The RealTime PCR assay is a highly sensitive test and has demonstrated high levels of reproducibility both within the laboratory and compared with external laboratories.^2^^,^^23^^,^^24^ By pooling samples, programs can save time and resources, and pooling appears to provide adequate precision even at low prevalence levels.^25^ Current PCR platforms such as Abbott m2000 can test up to 480 samples per run, and therefore could provide results in time to allow programmatic decision making.

Monitoring of ocular *C. trachomatis* infection alongside clinical indicators at the programmatic scale was possible and demonstrated that chlamydial transmission remained throughout much of the Amhara region. Districts with both high TF and infection should be immediate targets for enhancements to the SAFE strategy to reduce the force of infection and to reach elimination as a public health problem more quickly.

## Supplemental Material


Supplemental materials
