# Supplementary material for: Prevalence of Ocular Chlamydia trachomatis Infection in Amhara Region, Ethiopia, after 8 Years of Trachoma Control Interventions
Source: Am J Trop Med Hyg. 2023 Jan 9;108(2):261–7. doi: 10.4269/ajtmh.22-0535 (PMC9896333; doi:10.4269/ajtmh.22-0535)
Supplement: Supplementary file 1 [file tpmd220535.SD1.pdf]

Supplemental Table 1. District-level estimates of TF and TI among children ages 1 to 9 years and district-level estimates of ocular *Chlamydia trachomatis* (*Ct*) among children ages 1 to 5 years, by zone, district, year and survey type, Amhara, Ethiopia 2014-2021.

**Zone**	**District**	**Year**	**Survey Type**	**TF (1-9 y)**	**TI (1-9 y)**	***Ct* (1-5 y)**
Awi	Ankesha	2016	TIS	16.9	0.8	0.54
Awi	Banja	2018	TIS	3.99	0.6	0
Awi	Chagni Town	2016	TIS	3.8	0.1	0.5
Awi	Dangila Town	2016	TIS	1.2	0.4	0
Awi	Dangila Zuria	2016	TIS	3.6	0.8	0
Awi	Fagita Lekoma	2016	TIS	11.7	1.8	0
Awi	Guagusa-Shikudad	2016	TIS	28.3	2.8	0.54
Awi	Guangua	2016	TIS	7	1.2	0
Awi	Injibara town	2015	TSS	1.5	0.7	0
Awi	Jawi	2016	TIS	4.9	2	0
Awi	Zigem	2016	TIS	16.4	5.5	0.53
East Gojjam	Aneded	2018	TIS	15.1	1.3	3.92
East Gojjam	Awabel	2018	TIS	11.33	0.5	2.06
East Gojjam	Baso Liben	2018	TIS	18.12	2.3	5.36
East Gojjam	Bibugn	2016	TIS	10.6	1	0
East Gojjam	Debay Tilatgin	2016	TIS	27.9	5.7	4.57
East Gojjam	Debre Eliase	2018	TIS	6.64	0.5	0
East Gojjam	Debre Markos Town	2016	TIS	5	0	0
East Gojjam	Dejene	2018	TIS	20.07	4.2	11.63
East Gojjam	Enarge	2018	TIS	16.98	2.5	1.47
East Gojjam	Enebssie Sar Midir	2018	TIS	16.27	1	0.24
East Gojjam	Enemay	2018	TIS	23.46	4	3.49
East Gojjam	Goncha	2016	TIS	24	3	6.08
East Gojjam	Gozamin	2018	TIS	5.3	0.2	0.86

East Gojjam	Huletu Eju Enessie	2018	TIS	13.42	0.8	0.82
East Gojjam	Machakel	2016	TIS	14.1	1.5	0
East Gojjam	Motta Town Admin.	2016	TIS	7.3	0.7	0
East Gojjam	Sedie	2018	TIS	4.06	0.4	0
East Gojjam	Shebel	2018	TIS	22.92	1.6	3.63
East Gojjam	Sinan	2016	TIS	3.9	0.3	1.16
North Gondar	Aderkay	2017	TIS	3.63	0.5	0
North Gondar	Alefa	2015	TIS	4.6	1.5	0
North Gondar	Beyeda	2018	TIS	7.1	0.2	0
North Gondar	Chilga	2015	TSS	3.1	0.8	0
North Gondar	Dabat	2021	TIS	23.35	4	7.927
North Gondar	Debark Town	2018	TIS	3.34	0.4	0
North Gondar	Debark Zuria	2018	TIS	16.2	1.1	3.07
North Gondar	Dembiya	2017	TIS	17.49	2	8
North Gondar	East Belessa	2015	TIS	34.9	5.2	1.02
North Gondar	Genda Wuha Town	2015	TSS	1.8	1.2	0
North Gondar	Gondar Town	2017	TIS	5.7	0.3	0
North Gondar	Gondar Zuria	2015	TIS	42.8	14.1	11.22
North Gondar	Janamora	2021	TIS	18.89	2.2	2.6
North Gondar	Lay Armachiho	2017	TIS	1.14	0.1	0
North Gondar	Metema	2017	TIS	1	0.2	0
North Gondar	Quara	2017	TIS	4.73	1.6	0
North Gondar	Tach Armchiho	2015	TSS	3.2	1.2	0
North Gondar	Takussa	2015	TIS	13.9	2.8	1.01
North Gondar	Tegede	2017	TSS	2.48	0.3	0
North Gondar	Telemt	2018	TIS	8.61	0.4	0
North Gondar	West Armachiho	2017	TIS	7.5	2.8	0
North Gondar	West Belessa	2019	TIS	34.9	3.9	8.24
North Gondar	Wogera	2015	TIS	30	11.8	12.12
North Shoa	Angolelanatera	2016	TIS	9.9	3.9	0.5

North Shoa	Ankober	2018	TIS	3.62	0	0
North Shoa	Antsokia Gemza	2016	TIS	17.8	6.6	7.25
North Shoa	Asagirt	2017	TIS	6.2	1.4	3.33
North Shoa	Basona Worana	2016	TIS	7.8	1.5	5.16
North Shoa	Berehet	2017	TSS	3.2	0.5	1.23
North Shoa	Debre Birhan Town	2016	TIS	10.6	3	0
North Shoa	Efrata Gidim	2018	TIS	8.46	0.6	6.25
North Shoa	Ensaro	2020	TIS	30.05	3.6	9.58
North Shoa	Gishe	2018	TIS	6.13	0.3	6.67
North Shoa	H/Mariam Kesem	2017	TIS	15.1	1	1.32
North Shoa	Kewot	2016	TIS	15.9	3	1.89
North Shoa	Menz-Gera	2016	TIS	8.7	1.6	0
North Shoa	Menz-Keya	2019	TIS	11.81	0.7	2.73
North Shoa	Menz-Lalo	2016	TIS	8	2.3	0
North Shoa	Menz-Mama	2016	TIS	7.9	2.5	0
North Shoa	Merhabete	2019	TIS	36.09	5.5	34.37
North Shoa	Mida Woremo	2019	TIS	29.44	4.1	16.52
North Shoa	Minjar Shenkora	2018	TIS	8.22	0.7	0
North Shoa	Mojana Wodera	2018	TIS	7.74	0.4	2.44
North Shoa	Moretna Jiru	2019	TIS	30.09	2.5	15.18
North Shoa	Shoarobit Town	2016	TIS	4.8	0.9	0
North Shoa	Siadebirnawayu	2017	TIS	17.4	2.2	0.62
North Shoa	Tarmaber	2016	TIS	33.9	5.3	9.09
North Wollo	Angot	2018	TIS	0.8	0.3	0
North Wollo	Ayinabugina	2018	TIS	42.74	4.9	27.5
North Wollo	Dawunt	2018	TIS	39.02	6.7	16.96
North Wollo	Gazo	2018	TIS	4.27	0.6	2.7
North Wollo	Gidan	2018	TIS	14.41	0.7	0
North Wollo	Gubalafto	2017	TIS	3	0	0
North Wollo	Habiru	2017	TIS	5.4	0.6	1.55

North Wollo	Kobo Town Admin	2016	TIS	5	1.2	0
North Wollo	Kobo Zuria	2018	TIS	10.41	0.6	3.03
North Wollo	Lalibela Town	2018	TIS	14.18	1.9	3.49
North Wollo	Lasta	2018	TIS	24.47	1	5.94
North Wollo	Meket	2018	TIS	37	4.7	17.95
North Wollo	Wadilla	2018	TIS	22.66	1.4	7.27
North Wollo	Woldia	2017	TIS	1	0.3	0
Oromia	Artuma Furse	2018	TIS	11.87	1.3	1.67
Oromia	Bati Rural	2017	TIS	9.9	1.2	0
Oromia	Bati Town	2017	TIS	3.3	0.6	0
Oromia	Dawa Chafa	2018	TIS	17.44	2.1	3
Oromia	Dawey Harawa	2019	TIS	13.73	1	1.9
Oromia	Jille Tumuga	2018	TIS	9.03	0.8	0
Oromia	Kemmissie	2017	TIS	1.8	0	0.61
South Gondar	Andabet/West Estie	2017	TIS	36.96	6.2	11.32
South Gondar	Debre Tabor town	2016	TIS	4.7	0.4	0
South Gondar	Dera	2014	TIS	29.1	5	2.66
South Gondar	East Estie	2015	TIS	20.7	3.7	3.41
South Gondar	Ebinat	2014	TIS	49.5	9.5	10.3
South Gondar	Farta	2015	TIS	32.6	6.7	26.09
South Gondar	Fogera	2014	TIS	35.6	4.9	3.32
South Gondar	Lay Gaynt	2015	TIS	47.8	9.6	3.26
South Gondar	Libo Kemkem	2014	TIS	35.8	7.3	1.47
South Gondar	Simada	2015	TIS	45.7	8.4	5.68
South Gondar	Tach Gaynt	2014	TIS	35.8	7.4	6.43
South Gondar	Woreta town	2015	TIS	5.7	0.4	1.47
South Wollo	Albuko	2017	TIS	8.9	0	0.77
South Wollo	Ambassel	2017	TIS	2	0.6	1.8
South Wollo	Argoba	2020	TIS	41.56	6.6	16.78
South Wollo	Borena/Debresinna	2017	TIS	13.1	2.6	0

South Wollo	Delanta	2016	TIS	38.6	12.7	22.08
South Wollo	Dessie Ketema	2017	TIS	1.9	0.6	0
South Wollo	Dessie Zuria	2018	TIS	5.79	0.4	0
South Wollo	Jamma	2019	TIS	16.7	1	6.7
South Wollo	Kalala	2019	TIS	24.57	1.7	6.44
South Wollo	Kalu	2018	TIS	9.23	0.5	0
South Wollo	Kombolcha	2018	TIS	2.81	0.5	0
South Wollo	Kutaber	2017	TIS	14.9	1.8	7.75
South Wollo	Legambo	2017	TIS	10.2	2.4	0
South Wollo	Legehida	2017	TIS	33.7	4.5	6.08
South Wollo	Mehal Sayient	2019	TIS	9.49	0.4	0
South Wollo	Mekidella	2019	TIS	23.48	0.6	11.76
South Wollo	Sayient	2017	TIS	17.1	1.3	0.67
South Wollo	Tehuledere	2017	TIS	2.7	0.1	0
South Wollo	Tenta	2017	TIS	20.1	1.9	2.96
South Wollo	Wogide	2019	TIS	25.6	1.4	8.88
South Wollo	Worebabo	2020	TIS	23.88	2.5	11.48
South Wollo	Woreilu	2017	TIS	18.9	4.1	7.38
Waghemra	Abergelle	2017	TIS	19.6	3.8	2.13
Waghemra	Dahanna	2017	TIS	55.3	13.7	19.34
Waghemra	Gazegibilla	2019	TIS	31.53	1.1	12.84
Waghemra	Sahalla Seyemt	2016	TIS	13.3	1.7	0
Waghemra	Sekota Ketema	2019	TIS	14.76	2.1	3.05
Waghemra	Sekota Zuria	2019	TIS	32.39	2.2	11.51
Waghemra	Tsagebji	2019	TIS	47.98	3.5	23.6
Waghemra	Ziqualla	2017	TIS	16.5	2	0
West Gojjam	Bahir Dar city	2016	TIS	12.3	3.4	0.5
West Gojjam	Bahir Dar Zuria	2016	TIS	23.9	4	0
West Gojjam	Bure Town (Ketema)	2016	TIS	32.3	3.4	2.11
West Gojjam	Bure Zuria	2016	TIS	31.9	10	12.28

West Gojjam	Dega Damot	2016	TIS	2	0.3	0
West Gojjam	Dembecha	2016	TIS	15	8.3	1.6
West Gojjam	Finot Selam town	2015	TSS	4.8	0.9	1.42
West Gojjam	Gonje Kollela	2015	TIS	32.1	5.1	2.47
West Gojjam	Jabi Tinan	2015	TIS	15.5	1.8	0
West Gojjam	Mecha	2016	TIS	5.1	0.4	0
West Gojjam	North Achefer	2016	TIS	18.8	1.4	0
West Gojjam	Quarit	2015	TIS	13.4	2.9	0
West Gojjam	Sekella	2016	TIS	9.6	1.6	0
West Gojjam	South Achefer	2015	TIS	6.7	0.8	0
West Gojjam	Wonberma	2016	TIS	32.8	4.8	8.51
West Gojjam	Yilmana Densa	2016	TIS	10.8	2.1	0
